# Associations Between Oral Health Related Outcomes and Electronic Cigarette Use in Young People: A Scoping Review

**DOI:** 10.1111/ipd.70090

**Published:** 2026-04-16

**Authors:** Jessica Tran, Hollie Bendotti, Moya Vandeleur

**Affiliations:** ^1^ School of Dentistry, Faculty of Health, Medicine and Behavioural Sciences The University of Queensland Herston Queensland Australia; ^2^ Thoracic Research Centre, Faculty of Health, Medicine and Behavioural Sciences The University of Queensland Queensland Australia; ^3^ School of Public Health, Faculty of Health, Medicine and Behavioural Sciences The University of Queensland Herston Queensland Australia; ^4^ Respiratory Research Group Murdoch Children's Research Institute Parkville Victoria Australia; ^5^ Department of Respiratory and Sleep Medicine Royal Children's Hospital Parkville Victoria Australia

**Keywords:** electronic cigarettes, oral health, scoping review, vaping, young people

## Abstract

**Background:**

Vaping is prevalent among youth, but its effects on oral health remain poorly understood. Exposure to harmful chemicals and nicotine during this critical developmental period raises concerns for long‐term oral health impacts.

**Aim:**

This scoping review synthesizes available evidence regarding oral health related outcomes of e‐cigarette use in youth.

**Design:**

An electronic search was conducted in MEDLINE (PubMed), CINAHL, Embase, and Web of Science for studies examining associations between vaping and oral health in people aged 12 to 24 years. Studies were limited to English, with no date restrictions. Title and abstracts were screened, with full‐text review by two independent reviewers. Data extraction was completed by one reviewer and reviewed by all team members. Results were reported according to PRIMSA‐ScR.

**Results:**

Seven studies met inclusion criteria. Associations between vaping and dental caries, xerostomia, gingival conditions, oral lesions, and fractured teeth were observed. Alterations to salivary and microflora composition were also reported. Despite these risks, knowledge of the adverse effects of vaping among youth was low.

**Conclusion:**

Although vaping is associated with various negative oral health‐related outcomes in young people, the evidence is limited. Longitudinal studies with objective clinical measures are required to further clarify harm in this population.

## Why This Paper Is Important to Pediatric Dentists

- E‐cigarette use in young people may contribute to xerostomia, dental caries, and oral lesions.
- Knowledge of the oral health effects of vaping in young people is limited.
- Longitudinal studies with objective measures are required to clarify the relationships between e‐cigarette use and oral health in young people.

## 1 Introduction

The use of electronic cigarettes (e‐cigarettes), colloquially known as vapes, has become increasingly prevalent. Marketed as a “safer” alternative to traditional cigarettes, the addition of flavorings, bright packaging and lower costs increases the appeal and uptake of vaping in young people who have never smoked [1, 2]. Despite the perception of reduced harm compared to combustible cigarette smoking, these battery‐operated devices produce an inhalable aerosol that contains various toxic chemicals and nicotine [3, 4]. Growing literature has documented the deleterious effects of e‐cigarettes and their toxins on respiratory, cardiovascular, and psychosocial health [5, 6]. However, due to its recent emergence, there has been insufficient time to fully assess the long‐term consequences.

The oral cavity is the first point of exposure and is highly susceptible to the adverse effects of e‐cigarettes [7]. Research in adult populations has demonstrated associations between vaping and various dental diseases including gingivitis, periodontitis, and dental caries [8, 9, 10]. The use of e‐cigarettes has also been shown to alter the physiochemical composition of saliva, reducing its antioxidant capacity [11]. Preliminary studies further suggest that vaping is implicated with modifications in the oral microbiome [12]. These alterations diminish the defensive mechanisms within the oral cavity, increasing susceptibility to infections and disease [12]. Emerging evidence also suggests the potential for vaping cessation to improve oral health outcomes [13].

Despite the increasing prevalence of vaping among children, adolescents, and young adults, there remains a paucity of literature focused on the oral health effects in this susceptible population [4]. A survey by the Australian Institute of Health and Welfare in 2023 found that 49% of individuals aged 18–24 years in Australia had used an e‐cigarette in their lifetime [4]. This was almost double the previously reported statistic of 26% in 2019 [4]. It was also noted in this report that 21% of young people were daily e‐cigarette users [4]. The lack of regulations and legislative requirements have meant that e‐cigarettes have become more accessible, with individuals using e‐cigarettes at earlier ages [14]. Exposure to the harmful chemicals and nicotine within e‐cigarettes during the critical developmental periods of childhood and adolescence raises concerns for the long‐term impact on the oral tissues.

This scoping review aims to synthezise the available evidence on the oral health related outcomes associated with e‐cigarette use in young people. Understanding the risks is important for the development of targeted healthcare recommendations, oral health promotion activities, and guidelines for clinical practice in this population.

## 2 Methods

This scoping review adhered to the Joanna Briggs Institute (JBI) methodology for scoping reviews and PRISMA extension for scoping reviews [15, 16]. A protocol was registered prospectively with Open Science Framework (September 26, 2024) [17].

### 2.1 Eligibility

This scoping review was limited to young people aged 12 to 24 years old as defined by the definition provided by the Australian Institute of Health and Welfare [18]. Studies inclusive of this population that assessed the relationships between oral health outcomes and e‐cigarette use were included. Oral health outcomes were defined as oral conditions, oral health behaviors and perceptions, and oral health related outcomes. Both self‐reported and clinical measures were included. Articles were restricted to English, and no limitations were imposed for year of publication. Systematic and narrative reviews, letters to the editor, case reports, and cell and animal studies were excluded.

### 2.2 Search Strategy

A comprehensive electronic search was completed on PubMed, CINAHL, Embase, and Web of Science on September 27, 2024. Search strategies were adapted for each database (Appendices S1–S4) and included the following key terms:

(“Electronic Nicotine Delivery Systems”[tiab] OR ENDS[tiab] OR Vape*[tiab] OR Vaping[tiab] OR “electronic cigarette*”[tiab] OR Vaping[Mesh] OR ecig*[tiab] OR e‐cig*[tiab]) AND (“Young people”[tiab] OR “Young adult*”[tiab] OR Child*[tiab] OR Adolescen*[tiab] OR Adolescent[Mesh] OR Teenag*[tiab]) AND (“Oral Health*”[tiab] OR dent*[tiab] OR Oral[tiab] OR “Oral Health”[Mesh] OR Dentistry[Mesh]).

### 2.3 Study Selection

Articles from each database were imported into EndNote 21 (Clarivate Analytics) and duplicates were removed. References were then uploaded to Covidence for screening. Title and abstracts were screened according to the criteria by one reviewer (J.T.). Full‐text review was completed by two independent reviewers (J.T. and H.B.) and reasons for exclusions were detailed. Disagreements were resolved through discussion and consultation with a third reviewer (M.V.). Attempts were made to contact study authors by email for studies where age ranges were unclear or data stratified by age were not presented.

### 2.4 Data Extraction and Synthesis

Data extraction was completed by one reviewer (J.T.), which was then reviewed and agreed on by all review authors. Data extraction template fields included author, year, title, country, study aim, population (age of participants), sample size, oral health outcome, methods, key findings, and authors' conclusions. A narrative synthesis of the data was completed.

## 3 Results

**FIGURE 1:**
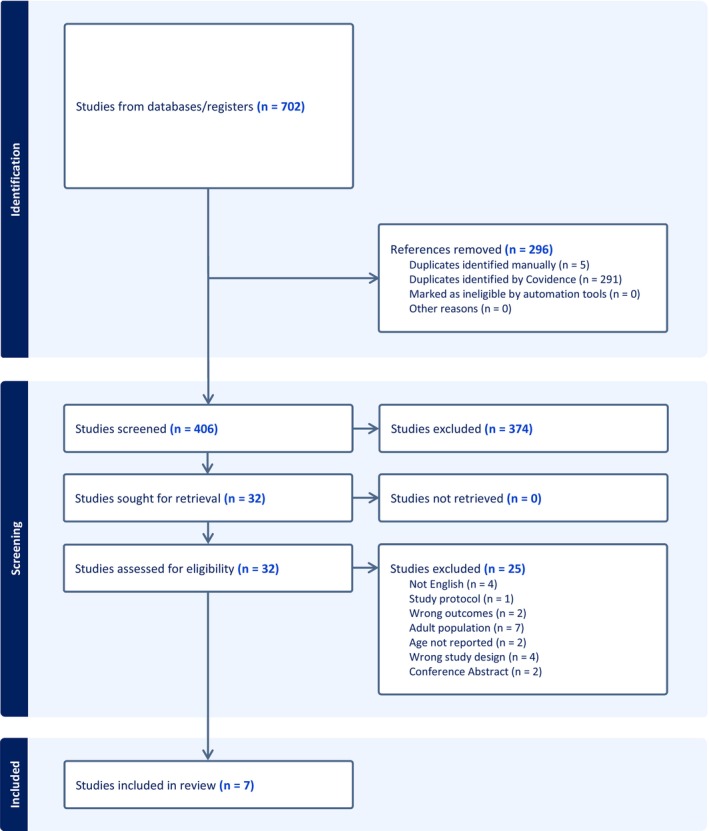
Preferred reporting items for systematic reviews and meta‐analyses flow diagram.

A total of 406 studies were identified for screening following removal of duplicates. After full‐text review, seven studies were deemed eligible for this review (Figure 1). Two studies had populations 1 year outside of the defined age range [19, 20]. Due to the paucity of literature, these studies were included as a majority of their data fell within the inclusion criteria.

### 3.1 Study Characteristics

A summary of the included studies is presented in Table 1. Five studies investigated the association between e‐cigarette use and the presence of oral health conditions. Of these studies, four used self‐report measures and one used an objective clinical measure [19, 21, 22, 23]. Oral conditions identified in e‐cigarette users included dental caries, xerostomia (dry mouth), gingival conditions, oral mucosa lesions, and fractured teeth [19, 20, 22, 23]. Two studies assessed the microbiological effects of e‐cigarette use on oral health [20, 25]. An additional study investigated knowledge and perceptions of vaping on oral health in young people [24]. No studies in this population evaluated the effect of e‐cigarette use on oral health behaviors.

**TABLE 1:** Summary of studies evaluating oral health outcomes of e‐cigarette use in young people.

Author, year	Country	Population	Sample size	Oral health outcome	Key findings
Akinkugbe, 2019 [21]	United States	12–17 years	13 650	Self‐reported provider diagnosis with dental problems	Dual use of e‐cigarettes and conventional cigarettes is associated with diagnosis of dental problems No associations were observed for e‐cigarettes alone
Alade et al., 2022 [19]	Nigeria	11–23 years	2870	Self‐reported oral lesions	Dry mouth, gingival inflammation and oral ulcers were significantly associated with e‐cigarette use Change in taste was associated with dual use
Alyaseen & Aldhaher, 2024 [20]	Iraq	18–25 years	90	Dental caries experience and salivary biomarker detection	Dental caries experience was higher in e‐cigarette group There were significant differences in Glucosyltransferase B and secretory Immunoglobulin A levels in saliva of e‐cigarette users
Chaffee et al., 2023 [22]	United States	9th and 10th grade students	976	Self‐reported measures of xerostomia	Xerostomia was not independently associated with e‐cigarette use
Cho, 2017 [23]	South Korea	7th to 12th grade students	65 528	Self‐reported gingival pain/bleeding, tongue and/or inside‐cheek pain, and cracked or broken tooth in past 12 months	E‐cigarette use increased the odds of tongue and/or inside‐cheek pain and having a cracked or broken tooth No associations between e‐cigarette use and gingival pain and/or bleeding
Hang et al., 2023 [24]	New Zealand	16–24 years	237	Knowledge and perceptions of EC use on oral health and self‐reported oral health status	51% thought that the mouth could be adversely affected by vaping Most frequently identified oral health risk was dry mouth 76% were happy to receive vaping related information from oral health professionals
Tishchenko et al., 2022 [25]	Ukraine	14–18 years	60	Bacterial colonization	Increase in species diversity and total bacterial density in e‐cigarette group 2.8 times decrease in resident microflora 5.7 times increase in number of *C. albicans*

### 3.2 Oral Health Outcomes

#### 3.2.1 Knowledge and Perceptions

A cross‐sectional study conducted in New Zealand was the only study to evaluate the knowledge and perceptions of e‐cigarette use on oral health in young people [24]. In this population, only 51% of respondents believed that the oral cavity could be adversely affected by vaping [24]. This was lower when compared to other organs including the lungs (96.9%), brain (58.6%), and heart (55.7%) [24]. Dry mouth was perceived to be the main oral health risk [24]. However, many participants indicated that they were unsure regarding the oral health risks associated with e‐cigarette use [24]. Although most (93.9%) reported they had never asked dental practitioners for vaping‐related information, participants were moderately willing to discuss e‐cigarette use with a dental professional [24].

#### 3.2.2 Self‐Reported Measures

A survey evaluating self‐reported oral lesions associated with vaping in youth in Nigeria found that gingival inflammation (AOR: 1.51, 95% CI: 1.00–2.26, *p* = 0.05), oral ulcers (AOR: 1.89, 95% CI: 1.02–3.49, *p* = 0.04) and dry mouth (AOR: 1.96, *p* = 0.01) were all significantly associated with e‐cigarette use [19]. Altered taste was not independently associated with e‐cigarette use (AOR: 1.62, 95% CI: 0.96–2.18, *p* = 0.07) but was found to be significant in dual users of combustible cigarettes and e‐cigarettes (AOR: 3.27, 95% CI: 2.32–4.61, *p* < 0.01) [19]. Results from this study were corroborated by a cross‐sectional study in Californian adolescents [22]. This study used a global measure of xerostomia in conjunction with the validated Shortened Xerostomia Inventory (SXI) to assess the associations of xerostomia with e‐cigarette use [22]. In this cross‐sectional study, dry mouth was positively associated with vaping (*p* < 0.01) [22]. When compared with individuals that had not used e‐cigarettes in the previous 30 days, use of e‐cigarettes for at least 6 days within a 30‐day period was associated with greater reports of frequently or always experiencing dry mouth (*p* < 0.01) [22]. Frequency of use did not influence the increased reporting of xerostomia [22]. Following covariate adjustment however, e‐cigarette use and xerostomia were no longer independently associated (AOR: 1.00, 95% CI: 0.55–1.80, *p* = 0.99) [22].

Contrary to findings by Alade et al., a large cross‐sectional study in a South Korean cohort of middle and high school students did not find any associations with e‐cigarette use and gingival pain and/or bleeding [19, 23]. Daily e‐cigarette use was, however, significantly associated with increased odds of tongue and/or inside cheek pain (AOR: 1.54, 95% CI: 1.05–2.26, *p* < 0.05) and cracked or broken teeth (AOR: 1.65, 95% CI: 1.19 –2.27, *p* < 0.01) [23]. Specifically, cracked and/or broken teeth were significantly associated with nicotine‐containing e‐cigarettes (AOR: 1.37, 95% CI: 1.15–1.63, *p* < 0.01) [23]. Nicotine‐free cigarettes were significantly associated with tongue and/or inside‐cheek pain (AOR: 1.56, 95% CI: 1.07–2.28, *p* < 0.05) [23]. Findings from a US sample of adolescents did not find any associations between self‐reported provider diagnosis of dental problems and e‐cigarette use, only dual use [21].

#### 3.2.3 Clinical Measures

A study by Alyaseen and Aldhaher evaluated the effect of e‐cigarette use on dental caries in relation to salivary biomarkers Glucosyltransferase B and Secretory Immunoglobulin A in a group of young adults [20]. Dental caries were diagnosed by oral examination with decayed, missing and filled teeth recorded [20]. When compared to the control, dental caries experience was significantly higher in the e‐cigarette group (*p* < 0.001) [20]. There were also significant differences observed in Glucotransferase B (*p* < 0.001) and secretory Immunoglobulin A levels (*p* < 0.001) in unstimulated saliva [20]. A positive correlation was also reported between Glucosyltransferase B (*p* = 0.001) and secretory Immunoglobulin A levels (*p* = 0.001) and dental caries [20].

Additionally, a study from Ukraine compared the microbiome composition of e‐cigarette users to combustible cigarette users and nonusers [25]. There was an increase in species diversity and total bacterial density in the e‐cigarette group when compared to the control [25]. Opportunistic Staphylococcal and Streptococcal flora representatives were increased in e‐cigarette users, with the most common species being *S. pneumoniae, S. mitis, and X. xerosis* [25]. Compared to the control, there was a decrease in resident microflora *S. mitis* representatives in the vaping group [25]. There was also a 5.7 times increase in the number of *Candida albicans* (*C. albicans* ) in the e‐cigarette group [25].

## 4 Discussion

The findings of this review provide an overview of the current literature on the associations between oral health related outcomes and e‐cigarette use in young people. Most studies identified in this review used self‐reported oral health outcomes, limiting the interpretation of the results. A main disadvantage of these studies is that they are subject to recall bias and misreporting [26]. Participants may provide exaggerated answers or underreport results due to social desirability bias which may over or underestimate their true effects. This highlights the need for stronger evidence through studies that utilize objective clinical measures. Majority of studies also did not explicitly state whether e‐cigarettes contained nicotine, marijuana, or were nicotine‐free, further limiting the ability to compare findings. Although the reliability of evidence may be low from these studies, e‐cigarette use has been implicated with dental caries, xerostomia, gingival conditions, oral mucosa lesions, and fractured teeth in this population [19, 20, 22, 23].

Xerostomia was the most reported oral health outcome associated with e‐cigarette use in young people [19, 22, 24]. The condition was perceived by adolescents as the most frequently identified oral health risk associated with vaping [24]. Furthermore, studies in a Nigerian and Californian cohort reported associations between xerostomia and e‐cigarette use [19, 22]. The consistency in findings across multiple populations suggests that dry mouth may be a prominent oral health issue for young e‐cigarette users. It should be noted that following adjustment for covariates including gender, ethnicity, asthma, physical activity, and previous 30‐day alcohol use, Chaffee et al. found that the association between e‐cigarette use and dry mouth was no longer significant [22]. This suggests that there are likely other factors including environmental and lifestyle variables that may contribute to these outcomes. Xerostomia is also commonly reported in adult populations, with adult e‐cigarette users also being more likely to experience dry mouth compared to combustible cigarette users [27]. Individuals with xerostomia have a reduced salivary flow and buffering capacity, which increases their susceptibility to caries, gingival conditions and other oral infections [28, 29, 30]. Future studies should be conducted with objective measures of xerostomia such as salivary flow rate to clarify its relationship to e‐cigarette use in this population.

Mixed results were identified in relation to gingival status. Alade et al. found significant associations between gingival inflammation and vaping [19]. Conversely, findings from Cho indicated no significant relationship between gingival pain or bleeding with e‐cigarette use [23]. Gingivitis can often go unrecognized in individuals with noticeable signs such as pain and bleeding only presenting with further disease progression [31]. Individuals with inadequate oral hygiene routines are unlikely to notice these milder symptoms themselves unless diagnosed by a dental professional. Adolescents are also less likely to take notice of their oral health, and it is likely that gingival symptoms were underreported in this cohort and do not reflect their true prevalence [32]. This discrepancy may also be explained by differences in demographics, variability in vaping habits, and cultural differences in oral health behaviors. Explanations for conflicting results may also be due to the varying compositions of e‐cigarettes. In the same study by Cho, nicotine‐containing e‐cigarettes were more strongly associated with cracked and broken teeth, and tongue and inside‐cheek pain were associated with nicotine‐free e‐cigarettes [23]. This indicates that varying components within e‐cigarettes likely affect oral health through different mechanisms. The contents of commercially available e‐cigarettes are highly variable [33]. Therefore, predicting their effects on different dental diseases continues to be a challenge.

Although a study in an American cohort did not find any independent associations between e‐cigarette use and provider‐diagnosed dental problems, it was limited by various factors [21]. Oral health outcomes were grouped together and associations with specific dental diseases may have been masked [21]. Furthermore, this study required participants to report provider diagnosed dental problems which omitted the possibility of reporting undiagnosed dental issues [21]. Symptomatic attendance and lack of access which have been linked with poorer oral health outcomes were not considered in this measure [34, 35]. However, this study did find associations with dual use of electronic and combustible forms of smoking [21]. This highlights the potential compounding effects of dual use in young people.

Although studies evaluating oral health through objective measures were limited by sample size, preliminary evidence provides insight into the potential effects of vaping on salivary and oral microbiome composition [20, 25]. Alyaseen and Aldhaher's research on dental caries found that e‐cigarette users had a higher experience of caries when compared to nonusers [20]. A significant difference in key salivary proteins Glucosyltransferase B and secretory Immunoglobulin A was also found between these groups [20]. Glucosyltransferases are produced by *Streptococuccus mutans* and play a central role in cariogenesis through glucan formation, whilst Secretory Immunoglobulin A contributes to mucosal immune defense against biofilm‐associated bacteria [20]. These markers reflect cariogenic activity and changes in host immune response. The positive correlation between these biomarkers and dental caries suggests that vaping may have a role in altering salivary composition, increasing the risk of caries in this population [20]. The study in a Ukrainian group of adolescents also emphasized the microbiological effects of e‐cigarette use in the oral cavity [25]. E‐cigarette users demonstrated increased abundance of opportunistic pathogens such as the *Staphylococcus* and *Streptococcus* species [25]. These species have been implicated in oral conditions such as angular cheilitis, mucositis, and caries [36, 37]. Furthermore, there was a significant increase in *C. albicans* , suggesting e‐cigarette use may create an environment conducive to fungal overgrowth [25]. Studies in conventional cigarette smokers have also reported microbial changes, with increases in *Streptococcus* species and depletion of Proteobacteria [38]. These findings are of concern considering the role of these pathogens in infections and systemic conditions [39, 40].

Despite research indicating various associations between oral health and vaping in young people, evidence regarding knowledge of the adverse effects is limited in this population [24]. The study by Hang et al. found that only around half of the participants believed that e‐cigarette use could affect the oral cavity, contrasting with more commonly recognized risks to the lungs, heart, and brain [24]. This finding suggests that education regarding the adverse effects of vaping is lacking [24]. Although respondents expressed a willingness to discuss e‐cigarette use with an oral health practitioner, many had not done so [24]. This emphasises that the opportunity for health promotion and advocacy within the dental setting may be underutilized. Research has indicated that oral health professionals demonstrated a poor working knowledge of alternative nicotine products and rates of use in their patients [41]. Given the limited but emerging evidence of e‐cigarette use and its oral health effects, it is likely many dental professionals do not feel equipped or confident in discussing e‐cigarette use and cessation [42]. Further education and training on e‐cigarette use and its oral health implications may encourage practitioners to engage in more constructive conversations with their patients on this topic.

Currently the scope of research regarding the adverse effects of e‐cigarette use on oral health in young people is still limited. Cases of maxillofacial injuries from e‐cigarette explosions and fatal carcinomas have been reported but are not well understood [43, 44]. In vitro studies have assessed the effects of vaping on dental esthetics, demonstrating a significant color difference post e‐cigarette exposure [45]. However, these findings are yet to be clarified in patient populations. Many studies included in this review had a cross‐sectional study design where causality could not be confirmed [19, 21, 22, 23, 24]. Longitudinal research would help to clarify the relationships between e‐cigarette use and different oral health outcomes. To reduce the risk of reporting bias, studies should consider the use of more objective clinical measures of oral health including salivary flow tests. As no studies assess oral health behaviors in this population, it also limits the capacity to fully comprehend the way e‐cigarette use impacts oral health.

Limitations of this review include selection bias due to the language restrictions imposed. There were several studies identified in the full‐text review which appeared to be relevant but were not written in English or translated [46, 47, 48]. Furthermore, the inclusion criteria of 12–24 years had meant that some studies were excluded despite their key population being young people. Studies in university students were often excluded due to the age range.

Although evidence is still emerging, available research suggests that e‐cigarette use in young people may contribute to oral conditions such as xerostomia, dental caries, and oral lesions. Preliminary evidence also suggests that vaping can also alter the saliva and oral microbiome composition in young people, placing them at a higher risk of dental caries and fungal infections. Despite these adverse effects, knowledge in young people is still limited, highlighting the need for better education and prevention strategies. Further longitudinal research with objective clinical measures is required to help clarify the relationships between e‐cigarette use and oral health in younger populations.

## Author Contributions

J.T., H.B., and M.V. conceived the ideas. J.T. collected the data. J.T., H.B., and M.V. analysed the data. J.T. prepared original draft. J.T., H.B., and M.V. reviewed and edited.

## Funding

The authors have nothing to report.

## Conflicts of Interest

The authors declare no conflicts of interest.

## Supporting information


**Appendix S1:** MEDLINE (PubMed) Search Strategy.
**Appendix S2:** CINAHL Search Strategy.
**Appendix S3:** Embase (Elsevier) Search Strategy.
**Appendix S4:** Web of Science Search Strategy.

## Data Availability

The data that supports the findings of this study are available in the Supporting Information of this article.
