# Supplementary material for: Associations Between Oral Health Related Outcomes and Electronic Cigarette Use in Young People: A Scoping Review
Source: Int J Paediatr Dent. 2026 Apr 16;36(4):577–84. doi: 10.1111/ipd.70090 (PMC13278668; doi:10.1111/ipd.70090)
Supplement: Supplementary file 1 — Appendix S1: MEDLINE (PubMed) Search Strategy. Appendix S2: CINAHL Search Strategy. Appendix S3: Embase (Elsevier) Search Strategy. Appendix S4: Web of Science Search Strategy. [file IPD-36-577-s001.docx]

**Appendices**

**Appendix 1. MEDLINE (PubMed) Search Strategy**

("Electronic Nicotine Delivery Systems"[tiab] OR ENDS[tiab] OR Vape*[tiab] OR Vaping[tiab] OR "electronic cigarette*"[tiab] OR Vaping[Mesh] OR ecig*[tiab] OR e-cig*[tiab])

AND

("Young people"[tiab] OR "Young adult*"[tiab] OR Child*[tiab] OR Adolescen*[tiab] OR Adolescent[Mesh] OR Teenag*[tiab])

AND

("Oral Health*"[tiab] OR dent*[tiab] OR Oral[tiab] OR "Oral Health"[Mesh] OR Dentistry[Mesh])

**Appendix 2. CINAHL Search Strategy**

((TI "Electronic Nicotine Delivery Systems" OR AB "Electronic Nicotine Delivery Systems") OR (TI Vape* OR AB Vape*) OR (TI Vaping OR AB Vaping) OR TI "electronic cigarette*" OR AB "electronic cigarette*" OR (MH Vaping+) OR (TI ecig* OR AB ecig*) OR (TI e-cig* OR AB e-cig*))

AND

((TI "Young people" OR AB "Young people") OR (TI "Young adult*" OR AB "Young adult*") OR (TI Child* OR AB Child*) OR (TI Adolescen* OR AB Adolescen*) OR (MH Adolescence+) OR (TI Teenag* OR AB Teenag*))

AND

((TI "Oral Health*" OR AB "Oral Health*") OR (TI dent* OR AB dent*) OR (TI Oral OR AB Oral) OR (MH "Oral Health+") OR (MH Dentistry+))

**Appendix 3. Embase (Elsevier) Search Strategy**

('Electronic Nicotine Delivery Systems':ti,ab OR ENDS:ti,ab OR Vape*:ti,ab OR Vaping:ti,ab OR 'electronic cigarette*':ti,ab OR Vaping/exp OR ecig*:ti,ab OR e-cig*:ti,ab)

AND

('Young people':ti,ab OR 'Young adult*':ti,ab OR Child*:ti,ab OR Adolescen*:ti,ab OR Adolescent/exp OR Teenag*:ti,ab)

AND

('Oral Health*':ti,ab OR dent*:ti,ab OR Oral:ti,ab OR Dentistry/exp OR 'dental health'/exp)

**Appendix 4. Web of Science Search Strategy**

((TI="Electronic Nicotine Delivery Systems" OR AB="Electronic Nicotine Delivery Systems") OR (TI=Vape* OR AB=Vape*) OR (TI=Vaping OR AB=Vaping) OR (TI="electronic cigarette*" OR AB="electronic cigarette*") OR (TI=ecig* OR AB=ecig*) OR (TI=e-cig* OR AB=e-cig*))

AND

((TI="Young people" OR AB="Young people") OR (TI="Young adult*" OR AB="Young adult*") OR (TI=Child* OR AB=Child*) OR (TI=Adolescen* OR AB=Adolescen*) OR (TI=Teenag* OR AB=Teenag*))

AND

((TI="Oral Health*" OR AB="Oral Health*") OR (TI=dent* OR AB=dent*) OR (TI=Oral OR AB=Oral))
